# Clinical utility of the BioFire FilmArray Blood Culture Identification panel in the adjustment of empiric antimicrobial therapy in the critically ill septic patient

**DOI:** 10.1371/journal.pone.0254389

**Published:** 2021-07-09

**Authors:** Roxanne Rule, Fathima Paruk, Piet Becker, Matthew Neuhoff, Julian Chausse, Mohamed Said

**Affiliations:** 1 Department of Medical Microbiology, University of Pretoria, Pretoria, South Africa; 2 Tshwane Academic Division, National Health Laboratory Service, Pretoria, South Africa; 3 Department of Critical Care, Steve Biko Academic Hospital, Pretoria, South Africa; 4 Department of Critical Care, University of Pretoria, Pretoria, South Africa; 5 Department of Biostatistics, University of Pretoria, Pretoria, South Africa; Hong Kong Children’s Hospital, HONG KONG

## Abstract

Sepsis and septic shock are key contributors to mortality in critically ill patients and thus prompt recognition and management thereof is central to achieving improved patient outcomes. Early initiation of appropriate antimicrobial therapy constitutes a crucial component of the management strategy and thus early identification of the causative pathogen is essential in informing antimicrobial therapeutic choices. The BioFire FilmArray blood culture identification (BCID) panel is a US Food and Drug Administration (FDA) approved rapid, multiplex polymerase chain reaction assay for use on positive blood cultures. This study evaluated its clinical utility in the intensive care unit (ICU) setting, in terms of amendment of empiric antimicrobial therapy in critically ill patients with sepsis. The assay proved useful in this setting as final results were made available to clinicians significantly earlier than with conventional culture methods. This, in turn, allowed for modification of empirical antimicrobial therapy to more appropriate agents in 32% of patients. Additionally, the use of the BioFire FilmArray BCID panel permitted the prompt implementation of additional infection prevention and control practices in a sizeable proportion (14%) of patients in the study who were harbouring multidrug resistant pathogens. These findings support the use of the BioFire FilmArray BCID panel as a valuable adjunct to conventional culture methods for the diagnosis and subsequent management of critically ill patients with sepsis.

## Introduction

In the critical care setting sepsis is not uncommon and contributes to significant morbidity and mortality, with mortality rates ranging from 30–80% [1–6]. As many as thirty to forty percent of all cases of sepsis are attributed to blood stream infections (BSIs) [2]. As such, comprehensive therapy which includes appropriate and timely administration of antimicrobial agents is crucial.

The Surviving Sepsis Campaign Guidelines recommend the administration of appropriate empiric intravenous antimicrobial therapy within one hour of making a diagnosis of sepsis; with blood cultures being obtained prior to the administration of the antimicrobial agents in order to optimise the yield of pathogen detection [7]. Inappropriate or inadequate dose and/or frequency of antibiotics has proven to be significantly associated with mortality in numerous studies, with documented mortality rates ranging between 10 to almost 40% [2,8,9]. A one-day prevalence study conducted by Paruk et al. in South African private and public ICUs in 2012 demonstrated that 54.9% of the patients who received empiric antibiotics, were inappropriately or inadequately treated [8]. The mortality rate associated with inappropriate antimicrobial therapy in the study was 27% (p = 0.01) [8]. Empiric antimicrobial therapy relies on pathogen identification for confirmation of its appropriateness or modification to the most suitable choice. Conventional/current blood culture techniques suffer from prolonged turn-around times, often contributing to delays in initiation of appropriate antimicrobial therapy. These limitations in current laboratory methods, together with the results demonstrated in the above studies, emphasise the crucial need for rapid and accurate diagnostic modalities in the workup of patients with BSIs. Early identification of the causative pathogens could potentially expedite appropriate antimicrobial therapy and improve patient outcomes.

The need for laboratory diagnostic tools that identify pathogens promptly in the scenario of sepsis has served as an impetus for molecular assays fast becoming useful adjuncts to routine laboratory processing. The BioFire FilmArray Blood Culture Identification (BCID) Panel (BioFire Diagnostics LLC, Salt Lake City, UT, USA) is one such tool. It is a multiplex real-time polymerase chain reaction (PCR) assay approved by the U.S. Food and Drug Administration (FDA) for use on positive blood cultures [10]. This assay has the ability to detect a total of 24 commonly encountered bacterial and fungal organisms, including *Staphylococcus* species, *Staphylococcus aureus*, *Streptococcus* species, *Streptococcus pneumoniae*, *Streptococcus pyogenes*, *Streptococcus agalactiae*, *Enterococcus* species, *Listeria monocytogenes*, *Enterobacterales*, *Acinetobacter baumannii*, *Enterobacter cloacae* complex, *Escherichia coli*, *Klebsiella pneumoniae*, *Klebsiella oxytoca*, *Proteus* species, *Pseudomonas aeruginosa*, *Serratia marcescens*, *Haemophilus influenzae*, *Neisseria meningitidis*, *Candida albicans*, *Candida glabrata*, *Candida parapsilosis*, *Candida krusei* and *Candida tropicalis* [10]. Further, the BioFire FilmArray BCID panel is able to detect three antimicrobial resistance genes, including *mecA* (encoding for methicillin resistance in staphylococci), *vanA/B* (encoding for vancomycin resistance in enterococci) and *bla*_*KPC*_ (encoding for carbapenemase production in *Enterobacterales*, *P*. *aeruginosa* and *A*. *baumannii*) [10].

This study aimed to evaluate the clinical utility of the BioFire FilmArray BCID panel when used as part of the diagnostic workup for critically ill patients with sepsis. To our knowledge this is the first study conducted in South Africa to assess the impact of the BioFire FilmArray BCID panel in the context of modifying the empirically initiated antimicrobial therapy in critically ill septic patients.

## Materials and methods

### Study design and setting

This prospective, interventional study was conducted at Steve Biko Academic Hospital, Pretoria, South Africa and the National Health Laboratory Service (NHLS), Tshwane Academic Division (TAD), Microbiology laboratory, Pretoria, South Africa. The study period included a total of ten consecutive months, between September 2019 and July 2020. This study was approved by the University of Pretoria Faculty of Health Sciences Research Ethics Committee (ethics reference number 246/2019). The ethics committee waived the need for informed consent from the critically ill patients whose blood cultures were included in the study.

### Blood culture specimens

Blood cultures submitted from patients admitted to five adult ICUs (surgical and trauma ICU, medical ICU, cardiothoracic ICU, coronary ICU and neurosurgery ICU) where sepsis was suspected comprised the study screening sample. All positive blood culture bottles (BacT/ALERT FAN Plus aerobic or anaerobic bottles ([bioMérieux, Marcy I’Etoile, France]) were processed with the BioFire FilmArray BCID panel as well as conventional culture methods. The sampling strategy included a consecutive case series of all patients with positive blood cultures that met the inclusion criteria. All blood culture specimen processing took place at the NHLS TAD Microbiology laboratory. Inclusion criteria included all blood cultures where the patients were over the age of 18 years, for whom blood cultures were taken during an episode of suspected sepsis and which subsequently flagged positive with microbial growth. Where paired blood culture bottles were received, only the bottle that flagged positive first was included in the study. Repeat positive blood cultures from the same patient within a 2-week time period were excluded from further analysis. The total sample size, therefore, includes 78 unique blood culture specimens obtained from critically ill, patients with sepsis.

### BioFire FilmArray Blood Culture Identification panel

Aliquots of the positive blood cultures were taken within one hour of the bottles flagging positive. These aliquots were processed independently by the principal study investigator (RR) with the BioFire FilmArray BCID panel. All specimen processing was done in accordance with manufacturer instructions [10]. This involved loading the relevant well of the BCID pouch with the provided hydration solution followed by 200 μL of broth from a positive blood culture bottle. This was then followed by the addition of the provided sample buffer into the sample injection well. The BCID pouch was then loaded into the BioFire FilmArray instrument (Release Version 2, Software Module Version: BioFire FilmArray FA Link UI 2.1.273.0). Thereafter, automated processing took place within the instrument, involving nucleic acid purification, multiplex PCR and lastly analysis of DNA melting curves to confirm and identify the presence of bacterial and fungal targets as well as antimicrobial resistance genes within the culture being tested.

### Conventional culture methods

Conventional culture methods were performed by the diagnostic laboratory staff in parallel to the BioFire FilmArray BCID panel for each patient specimen. Standard operating procedures were followed for processing and analysis of conventional cultures [11]. This entailed performing a direct Gram stain on each positive blood culture bottle. Thereafter, each bottle was subcultured onto solid agar media comprising 5% sheep blood agar, chocolate agar and MacConkey agar (Diagnostic Media Products [DMP], Johannesburg, South Arica). Depending on the Gram stain result, additional agar plates were inoculated to aid with the workup of each specimen. For Gram positive cocci, mannitol salt agar, bile esculin agar and DNAse agar (DMP, Johannesburg, South Africa) plates were also inoculated. Direct inoculation of the Vitek 2 (bioMérieux, Marcy I’Etoile, France) instrument was performed according to methodology published by Bamford et al (2010) [12] for all specimens yielding monomorphic Gram negative bacilli on gram stain. For Gram positive bacilli, an additional bile esculin agar plate was inoculated to facilitate earlier presumptive identification of *Listeria monocytogenes*. Where yeasts were detected on direct Gram stain, an additional Sabouraud agar (DMP, Johannesburg, South Africa) plate was inoculated. Inoculated agar plates were then incubated at 35°C ±2°C in 5% CO_2_ for a duration of 18–24 hours. In addition to the above agar plates, each anaerobic blood culture bottle was also inoculated onto a 10% sheep blood agar (DMP, Johannesburg, South Africa) plate that was incubated at 35°C ±2°C anaerobically for a total duration of 48 hours. All plates were inspected after the incubation period. Additional bench side and biochemical tests, such as oxidase, catalase and indole, were performed on colonies grown on subculture plates according to standard operating procedures [11]. Final species identification and antimicrobial susceptibility of the cultured organisms was performed with the Vitek 2 system. All *Enterobacterales* isolates with carbapenem non-susceptibility were further tested with the modified carbapenem inactivation method (mCIM) to confirm the presence of carbapenemase production. Antimicrobial susceptibility data was interpreted according to the CLSI 30^th^ edition M100 2020 breakpoints document [13].

### Measurements

For each specimen analysed, the following times were captured; time (hours) taken for the blood culture bottle to flag positive, time (hours) taken for the Gram stain result to be made available to treating clinicians and time (hours) for communication of final results generated by both conventional methods and the BioFire FilmArray BCID panel. Choice of empirical antimicrobial therapy before communication of the BioFire FilmArray BCID panel results as well as the intended choice of adjusted antimicrobial therapy after communication of the BioFire FilmArray BCID panel results was also recorded. This information was obtained from the clinician when the BioFire FilmArray BCID panel results were relayed. In addition, the indication for the blood culture in each patient was also obtained and recorded.

### Statistical analysis

Statistical analysis was performed with Stata (release version 15.1) (StataCorp, TX, USA) and MedCalc Version 19.2.1 (MedCalc Software Ltd, Ostend, Belgium). Conventional culture and the BioFire FilmArray BCID were compared with respect to time taken to be able to advise on appropriate antimicrobial therapy using a paired test. Time taken to advise on implementation of infection prevention and control practices for multidrug resistant organisms was also compared between the two methods. Whether adjustments in antimicrobial therapy were made in response to the BioFire FilmArray BCID result was also recorded. A sub-analysis for time taken to communicate results between the BioFire FilmArray BCID panel and conventional culture was performed for all patients in whom the BioFire FilmArray BCID panel had an impact on antimicrobial adjustment.

## Results

A total of 78 positive blood cultures from critically ill septic patients were included in this study. The majority (n = 39) of blood cultures were received from patients admitted to the surgical and trauma ICU. This was followed by the medical ICU (n = 20), neurosurgical ICU (n = 9), coronary ICU (n = 9) and finally cardiothoracic ICU (n = 1). Bact/ALERT FAN Aerobic Plus bottles comprised most of the bottles that flagged positive earliest in each blood culture pair (n = 44, 56.4%). The mean time to culture positivity was 16.9 hours (range 2 to 124 hours). Most of the specimens were obtained from male patients (n = 46, 59%). The mean age of the patients included in the study was 46 years (range 18 to 80 years).

### Time to communication of results

The mean time to communication of results generated by the BioFire FilmArray BCID panel was 4 hours and 6 minutes. This is in contrast to conventional methods that involved a mean time of 5 hours and 25 minutes for communication of the direct Gram stain findings and 2 days, 9 hours and 32 minutes for communication of final identification and antimicrobial susceptibility results. The mean difference in turn-around time between the BioFire FilmArray BCID panel and Gram stain was 46 minutes, with the BioFire result being faster (95% confidence interval [CI], -106–15 minutes, P = 0.20). The mean difference in turn-around time between the BioFire FilmArray BCID panel and final culture results was 51 hours and 17 minutes (95% CI, 3471–2683 minutes, P < 0.0001), implying that the BioFire FilmArray BCID panel was 2 days, 3 hours and 17 minutes faster than culture at generating a final report.

### Organisms identified

A total of 77 patients (98.8%) cultivated organisms on their positively flagged blood cultures. One blood culture bottle failed to have any evidence of organisms with both the BioFire FilmArray BCID panel and conventional culture methods, representing a false positive flag signal from the instrument. Most patients (n = 62, 79.5%) harboured monomicrobial blood cultures. A total of 93 organisms were cultured from the 78 patient blood cultures bottles included in the study. The most commonly encountered organisms in this study comprised the coagulase negative staphylococci (CoNS) with a prevalence of 25.8% (n = 24) followed by *K*. *pneumoniae* with a prevalence of 18.3% (n = 17) and *A*. *baumannii* with a prevalence of 11.8% (n = 11). Four patients had evidence of candidaemia. This included one patient with *C*. *glabrata*, one patient with *C*. *krusei* and two patients with *C*. *parapsilosis*. A total of six organisms were cultured that are not included in the BioFire FilmArray BCID panel, including one each of *Stenotrophomonas maltophilia* and *Acinetobacter ursingii*, both potentially significant Gram negative bacilli. The remaining four organisms, which are not on the BioFire FilmArray BCID panel, included *Bacillus* species, *Corynebacterium* species, *Faklamia hominis* and an anaerobe, namely *Propionibacterium acnes*. Table 1 summarises the list of organisms detected in this study that are included and excluded from the BioFire FilmArray BCID panel repertoire.

**Table 1:** Summary of organisms detected.

Organisms cultured that are included in the BCID panel repertoire (n = 87)	Total number identified in study
**Enterococcus species (n = 8)**	
*Enterococcus faecium*	3
*Enterococcus faecalis*	5
**Staphylococcus species (n = 28)**	
Coagulase negative staphylococci	24
*Staphylococcus aureus*	4
**Streptococcus species (n = 1)**	
*Streptococcus gallolyticus*	1
***Acinetobacter baumannii***	11
***Enterobacterales* (n = 33)**	
*Enterobacter cloacae* complex	5
*Enterobacter aerogenes*	1
*Escherichia coli*	3
*Klebsiella oxytoca*	2
*Klebsiella pneumoniae*	17
*Proteus*	1
*Serratia marcescens*	0
*Providentia stuartii*	1
*Morganella morgannii*	2
*Citrobacter freundii*	1
***Pseudomonas aeruginosa***	2
***Candida albicans***	0
***Candida glabrata***	1
***Candida krusei***	1
***Candida parapsilosis***	2
***Candida tropicalis***	0
**Organisms cultured that are not included in BCID panel repertoire (n = 6)**	
*Acinetobacter ursingii*	1
*Stenotrophomonas maltophilia*	1
*Corynebacterium* species	1
*Bacillus* species	1
*Faklamia hominis*	1
*Propionibacterium acnes*	1
**Total**	**93**

### Antimicrobial resistance

A total of seven patients grew staphylococci demonstrating methicillin resistance with corresponding *mecA* detection on the BioFire FilmArray BCID panel. One of these patients cultured a methicillin resistant *Staphylococcus aureus* (MRSA) on blood culture. The remaining six patients cultured coagulase negative staphylococci. No patients cultured vancomycin resistant enterococci (VRE). Of note, seven patients cultured carbapenem resistant *K*. *pneumoniae*, confirmed as carbapenemase producers with the mCIM test. None of these were positive for *bla*_*KPC*_ with the BioFire FilmArray BCID panel.

### Adjustment of antimicrobial therapy

**Fig 1:**
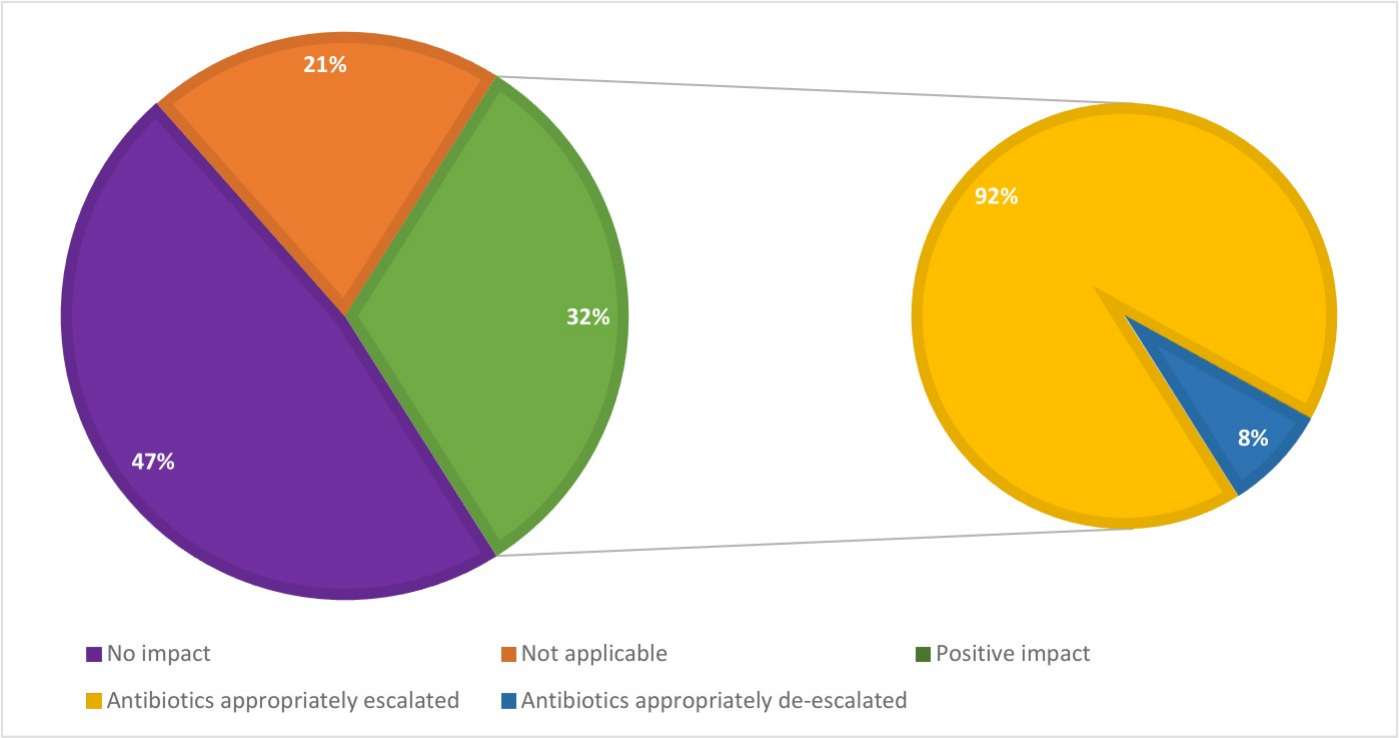
Impact of the BioFire FilmArray BCID panel on the adjustment of antimicrobial therapy.

Following discussion of results generated by the BioFire FilmArray BCID panel with the treating clinicians, antimicrobial therapy was adjusted for 25 patients (32%). Among these patients, 23 (92%) underwent appropriate escalation of antimicrobial therapy and the remaining 2 (8%) had their antimicrobial therapy appropriately de-escalated. The mean time to communication of results for the BioFire FilmArray BCID in the subgroup of patients who had appropriate adjustment of therapy was 3 hours and 33 minutes. The mean difference in turn-around time for the BioFire BCID compared to Gram stain and culture for patients in whom there was a positive impact on therapy was 1 hour, 37 minutes (P = 0.03, 95% CI, 10.0–183.2) and 56 hours, 55 minutes (P < 0.001, 95% CI, 2698.8–4130.7), respectively. This means that the BioFire FilmArray was, on average, one hour, 37 minutes faster at generating a result than the Gram stain and 2 days, 8 hours and 55 minutes faster than final culture results in this subgroup analysis. For 37 patients (47%), the BioFire FilmArray BCID had no impact on adjustment of therapy. In the remaining 16 patients (21%), adjustment of antimicrobial therapy could not be evaluated and was deemed not applicable as the BioFire FilmArray BCID panel failed to detect any organism targets in 3 of these patients and 13 patients demised before the BioFire FilmArray BCID results were available to the treating clinicians. Fig 1 defines the proportions of patients in whom the BioFire FilmArray had an impact on the adjustment of empirical antimicrobial therapy in this study.

### Infection prevention and control

In this study, a total of eleven (14.1%) multidrug resistant organisms, as defined by Magiorakos et al. [14], were detected by the BioFire FilmArray BCID panel, allowing for earlier implementation of additional infection prevention and control (IPC) measures including patient isolation. These comprised 10 *Acinetobacter baumannii* cases and one methicillin-resistant *Staphylococcus aureus* (MRSA) case. Given the local epidemiology comprising high rates of multi-drug resistant *Acinetobacter baumannii* isolates in the Pretoria region of South Africa, patients who had positive blood cultures with *A*. *baumannii* detected by the BioFire FilmArray BCID panel were managed as infection control alert cases requiring strict isolation and contact precautions, while awaiting final antimicrobial susceptibility test results. All *A*. *baumannii* cases detected in this study were subsequently confirmed to be multidrug-resistant with conventional culture methods. The mean time to communication of BioFire FilmArray BCID results for these important nosocomial pathogens was 4 hours and 11 minutes. The mean difference in turn-around time for communication of these results and subsequent advice on infection prevention and control measures for the BioFire FilmArray BCID panel compared to culture was 63 hours and 7 minutes (P = 0.003, 95% CI 4723.2–2851.5). This means that the BioFire FilmArray BCID panel was, on average, 2 days, 15 hours and 7 minutes faster than conventional culture for reporting IPC pathogens in this study.

## Discussion

Sepsis management necessitates a multipronged approach including early detection of the causative pathogen as well as timely administration of appropriate empiric antimicrobial therapy in order to avoid poor outcomes [5,15,16]. This renders rapid diagnostic assays as attractive tools to potentially add value to antimicrobial stewardship and improve outcomes in patients with sepsis. The BioFire FilmArray BCID panel has the potential to fulfil this role. Our findings demonstrate the clinical value of the BioFire FilmArray BCID panel in the management of critically ill patients with sepsis.

The impact of the BioFire FilmArray BCID panel was analysed for seventy-eight positive blood cultures from critically ill patients deemed to have sepsis. The time to communication of results to the treating clinician, generated by the BioFire FilmArray BCID panel and subsequent therapeutic advice was substantially faster than conventional culture methods. When comparing the BioFire FilmArray BCID panel to direct Gram stain, in our setting results were made available to clinicians at an average of 46 minutes earlier, potentially allowing for earlier adjustment of antimicrobial therapy, however this finding was not statistically significant (P = 0.20). The longer turn-around time to reporting Gram stain results in our setting rendered the BioFire FilmArray BCID panel more useful for prompt therapeutic decision making than conventional methods. This situation, however, may be unique to our setting due to batching of blood cultures for Gram staining with an automated stainer and is therefore, not generalisable. This shortcoming in the laboratory workflow has been highlighted to the management and retraining of staff was subsequently commenced. Compared to final, standardised culture results, the BioFire FilmArray BCID panel was significantly faster by an average of 2 days, 3 hours and 17 minutes (P < 0.0001). Similar observations have emerged from other studies [17–19]. Pardo et al (2016) found the BioFire FilmArray BCID panel to be substantially faster for both bacterial and fungal detection [17]. With regards to Gram positive bacteria, the BioFire FilmArray BCID results were available 24.7 hours before preliminary organism identification and 47.4 hours before final identification and susceptibility [17]. Confirmation of candidaemia with the BioFire FilmArray BCID panel was available 24.7 hours earlier than preliminary identification and 95.9 hours earlier than final identification and susceptibility [17]. Similarly, Payne et al (2018) demonstrated a much shorter turn-around time for the BioFire FilmArray BCID panel (2.2 hours) compared to standardised culture based methods (33.3 hours) [19].

The most commonly encountered organisms in this study were the coagulase negative staphylococci (prevalence 25.8%). Importantly, of the 24 patients that cultured CoNS, only three patients (12.5%) were deemed to have clinically significant bacteraemia. Two were being managed for infective endocarditis and one with angina pectoris and an unknown source of sepsis. All three patients had *mecA* detected with the BioFire FilmArray BCID panel, paving the way for early directed therapy with vancomycin. Among patients with contaminated blood cultures, the BioFire FilmArray BCID panel confers the benefit of earlier cessation of inappropriate antimicrobial therapy, an important factor in the era of multidrug resistance.

A limitation of the BioFire FilmArray BCID panel is its inability to detect organisms, other than CoNS, that are frequently labelled as skin contaminants (*Bacillus* species, diphtheroids and micrococci). Although not usually significant, these organisms are often the reason for blood culture bottles flagging positive and their identification may aid in appropriate cessation of empiric antibiotics. The high rate of non-significant CoNS together with the *Bacillus* species and *Corynebacterium* species cultures, translates to an alarmingly high blood culture contamination rate of 29.4% in this study, well above the accepted 3% contamination rate [11]. Contamination rates above 5% makes interpretation of positive blood cultures especially challenging to clinicians and often unnecessary and toxic antimicrobials are administered as a result [11]. Strategies to advise and educate clinicians on appropriate blood culture collection techniques are essential to adequately inform antimicrobial treatment choices.

Notably, one patient in this study cultured *Stenotrophomonas maltophilia*, an organism not included in the BioFire FilmArray BCID panel. *S*. *maltophilia* is a nosocomial pathogen increasingly associated with infection in critically ill patients [20]. Due to the inherent resistance to commonly used empiric antibiotics, such as the carbapenems, and the associated increased mortality risk to patients with *S*. *maltophilia* bacteraemia, it is critical to identify these organisms early in the disease course to ensure adequate treatment and outcomes [20]. Another troublesome pathogen not included in the BioFire FilmArray BCID panel repertoire is the emerging and multidrug resistant yeast, *Candida auris* [21]. *C*. *auris* poses a diagnostic challenge to laboratorians due to the inability of many phenotypic identification systems to distinguish it from other members of the *Candida haemulonii* complex, often contributing to prolonged turn-around times [21]. In addition, *C*. *auris* commonly contaminates patient environments and colonises skin surfaces, facilitating the emergence of outbreaks in the ICU setting [21].

In this study, no *Klebsiella pneumoniae* carbapenemase (KPC) producing Gram negative organisms were identified with the BioFire FilmArray BCID panel. While this enzyme is prevalent in other regions of the world, such as the United States of America (USA), it is rarely detected in South Africa [22,23]. According to the 2018 Annual GERMS-SA report, the most frequently encountered carbapenemases in *Enterobacterales* in South Africa are the OXA-48-like (60%) and New Delhi metallo-β-lactamases (NDM) (30%) [23]. No KPC producing *Enterobacterales* were reported during the surveillance period [23]. This limits the utility of the BioFire FilmArray BCID panel in the South African setting, as patients with sepsis due to carbapenemase producing *Enterobacterales* (CPE) will remain undetected with the use of the assay. Conventional culture methods and susceptibility testing is therefore still required to confirm carbapenem resistance in this setting.

Fortunately, a recent upgrade in the form of the BioFire FilmArray BCID2 panel overcomes the limitations listed above. The new panel has a more diverse range of organisms, allowing for detection of *S*. *maltophilia* and *C*. *auris* [24]. In addition, the repertoire of antimicrobial resistance gene targets has been expanded to include additional carbapenemase genes, such as *bla*_*NDM*,_ *bla*_*OXA-48-like*,_ *bla*_*IMP*_ and *bla*_*VIM*,,_ as well as a colistin resistance gene, *mcr-1*, and an extended spectrum beta-lactamase gene, *bla*_*CTX-M*_ [24].

In our setting, the BioFire FilmArray BCID panel clearly provided valuable information early and shaped management decisions. The panel allowed for earlier adjustment of antimicrobial therapy in 32% of patients. In the subgroup of patients who benefited from adjustment of antimicrobial therapy, the BioFire FilmArray BCID panel results were communicated a mean of 2 days, 8 hours and 55 minutes earlier than conventional culture methods. The majority of patients underwent escalation of antimicrobial therapy (addition of a carbapenem with or without colistin) for Gram negative sepsis. Two patients with yeasts detected with the BioFire FilmArray had treatment adjustments to more appropriate antifungal agents; one patient with *C*. *parapsilosis* not on antifungal therapy and another with *C*. *krusei* on fluconazole. The use of the BioFire FilmArray BCID panel in these patients allowed for earlier addition of an echinocandin when compared to conventional culture methods. Additionally, patients with Gram positive sepsis were also positively impacted by the use of the BioFire FilmArray BCID panel. In six patients, antimicrobial therapy was escalated to vancomycin following detection of MRSA, enterococci and clinically significant CoNS harbouring the *mecA* gene (as discussed above). One antibiotic naïve patient was initiated on cefazolin following detection of *mecA* negative *S*. *aureus*. The use of this panel also allowed for appropriate de-escalation of antimicrobial therapy in 8% of patients. This entailed stopping a carbapenem in a patient who was also receiving linezolid for suspected infective endocarditis. In this patient, the BioFire FilmArray BCID panel detected CoNS. Due to the clinical history, the treating clinicians opted to continue with linezolid monotherapy following the BioFire FilmArray BCID result. For a separate patient who was being treated for ventilator associated pneumonia (VAP) with meropenem and linezolid, the BioFire FilmArray BCID panel detected *K*. *pneumoniae* on this patient’s blood culture. The linezolid was thus stopped. Subsequent conventional culture methods yielded a carbapenemase producing *K*. *pneumoniae* in this patient. A similar impact on antimicrobial prescription practices has also been demonstrated in other studies [17,25–28]. A recent study conducted by Verroken et al (2019) found a similar impact to the present study, with 31.8% of 110 patients undergoing antimicrobial adjustment in response to BioFire FilmArray BCID results [26]. Most cases involved initiation of appropriate antimicrobial agents, followed by de-escalation and broadening of antimicrobial cover [26]. As in the present study, time to optimal antimicrobial therapy was drastically reduced with the BioFire FilmArray compared to conventional methods (4 hours 39 minutes versus 14 hours 41 minutes, respectively) [26].

A noteworthy proportion (n = 19, 24.4%) of patients harboured multidrug resistant organisms necessitating enhanced infection prevention and control practices in this study. This included 14.1% (n = 11) that were detected with the BioFire FilmArray BCID panel, comprising 10 *A*. *baumannii* cases and one MRSA case. The remaining 10.3% included seven non-KPC producing carbapenem resistant *Enterobacterales* (CRE) and one multidrug resistant *P*. *aeruginosa*. Turn-around time to advising isolation and contact precautions for the patients that had multidrug resistant organisms detected with the BioFire FilmArray BCID panel was substantially faster than with conventional culture methods. The mean time it took for patients to be isolated was 2 days, 15 hours and 7 minutes earlier when the BioFire FilmArray BCID panel was used. This potentially translates to a reduced likelihood of ongoing transmission of these particular organisms in the respective units as well as potentially reduced antimicrobial resistance in the hospital. The large proportion of CRE detected in this study highlights the need for rapid and accurate detection of these organisms to facilitate earlier IPC practices in infected patients. Unfortunately, the lack of incorporation of non-KPC carbapenemase enzymes in the BioFire FilmArray BCID panel repertoire limited the role that the assay played in preventing transmission of CRE in this study.

## Conclusion

Our findings support the use of the BioFire FilmArray BCID panel as an adjunct to conventional culture methods. The panel provided rapid results which permitted for appropriate adjustment of empirical antimicrobial therapy in a large proportion of patients. In addition, more stringent infection prevention and control measures were executed much sooner with the use of the assay. The rapidity of results generated by BioFire FilmArray BCID panel has the potential to translate to improved patient outcomes as well as aid in the prevention of emergence and transmission of antimicrobial resistance.

## Supporting information

S1 Data BioFire data. (XLSX)
